# The Tunneled Coronally Advanced Flap (TCAF) for Root Coverage Procedures: Rationale, Indications, and a Novel Papilla‐Based Decision Tree

**DOI:** 10.1111/jre.70147

**Published:** 2026-07-04

**Authors:** Lorenzo Tavelli, Shayan Barootchi

**Affiliations:** ^1^ Department of Oral Medicine, Infection, and Immunity, Division of Periodontology Harvard School of Dental Medicine Boston Massachusetts USA; ^2^ Center for Clinical Research and Evidence Synthesis in Oral Tissue Regeneration (CRITERION) Boston Massachusetts USA; ^3^ School of Dentistry Universidad Catolica de Santiago de Guayaquil (UCSG) Guayaquil Ecuador; ^4^ Department of Periodontics & Oral Medicine University of Michigan School of Dentistry Ann Arbor Michigan USA

**Keywords:** decision tree, gingival recession, papilla, root coverage, TCAF, tunneled coronally advanced flap

## Abstract

This technical report describes the clinical rationale for TCAF and introduces a papilla‐based decision tree to assist clinicians in selecting the most appropriate strategy (open vs. tunnel) for each individual papilla.

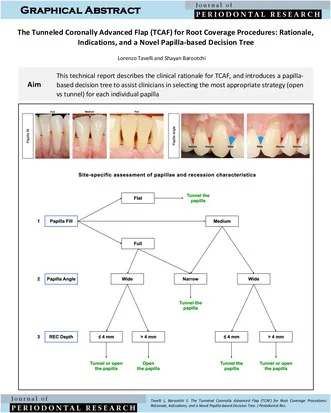

## 1 Introduction

Gingival recession is a highly prevalent condition that can pose both functional and esthetic challenges, particularly when located in the esthetic zone or associated with root sensitivity and plaque accumulation. Among the surgical techniques developed for root coverage, the Coronally Advanced Flap (CAF) and Tunnel (TUN) techniques have emerged as two (among many) of the most widely studied and commonly used, each with proven efficacy when applied appropriately [1, 2, 3, 4]. However, both approaches may face limitations in anatomically complex cases.

Recent literature emphasizes that the success of root coverage procedures is not solely determined by surgical technique, but also by anatomical factors including papilla dimensions, interproximal clinical attachment levels (CAL), recession depth, keratinized tissue (KT) width, and gingival thickness (GT) [2, 5, 6, 7, 8, 9]. These parameters often dictate the suitability and predictability of one approach over another and may significantly influence both short‐ and long‐term outcomes.

In this context, the Tunneled Coronally Advanced Flap (TCAF) has been developed as a surgical approach that integrates the principles of CAF, TUN, and others in a flexible and site‐specific manner with the aim of enhancing surgical outcomes, as well as patient and surgeon experience.

TCAF has emerged not as a new technique, but as a conceptual evolution in the surgical management of soft tissue defects—both around teeth and implants—offering a flexible, anatomy‐driven framework in which flap design is tailored to the specific characteristics of each site. Rather than following a fixed protocol, the clinician evaluates vital local anatomy, such as papilla morphology, recession depth, interproximal support, and tissue phenotype, to determine where to selectively incise, preserve, or tunnel a papilla. This site‐specific strategy enhances surgical access, improves flap mobility, facilitates graft insertion, stabilization, and ultimately increases predictability and outcomes across diverse clinical scenarios. Moreover, the approach is also intended to increase safety during the surgical procedure (by considering vital anatomical features), as well as increase clinician convenience (by avoiding unnecessary surgical steps).

This manuscript presents the clinical rationale for TCAF, explores anatomical factors that guide its design, and introduces a decision tree to assist clinicians in selecting the most appropriate root coverage strategy for each individual case, based primarily on the interproximal papillae and recession depth.

## 2 Rationale for the Use of the TCAF in Root Coverage Procedures

The efficacy and predictability of the CAF and TUN have been well demonstrated in the literature as the most predominant and well‐documented surgical designs in root coverage. These two approaches (also referred to commonly as the open vs. closed approach) encompass the majority (if not all) of other techniques.

When reviewing the published evidence relative to the efficacy of the two approaches (CAF and TUN), some studies show comparable results in terms of mean root coverage (mRC) [10, 11, 12, 13, 14, 15], while others show one or the other technique to be superior [16, 17, 18, 19, 20, 21, 22]. The compiling assessment of the evidence supports the notion that when applied with appropriate case selection (and at ideal sites) both CAF and TUN can yield similarly favorable outcomes, and that the short‐ and long‐term outcomes of root coverage procedures are mainly affected by other factors, such as CAL, KT width, GT, and the patient's adherence to atraumatic oral hygiene practices, among other variables [1, 7, 9, 12, 23, 24, 25].

Indeed, CAF and TUN are both known predictable and reliable approaches for root coverage procedures, and the choice of the approach should be based on the local anatomical characteristics of a defect. Key parameters that influence this decision may include the presence of interproximal attachment or papilla loss, black triangles, papilla width and height, recession depth, residual apical and lateral KT, gingival thickness, and the presence of frenula, among others (Figure 1).

**FIGURE 1:**
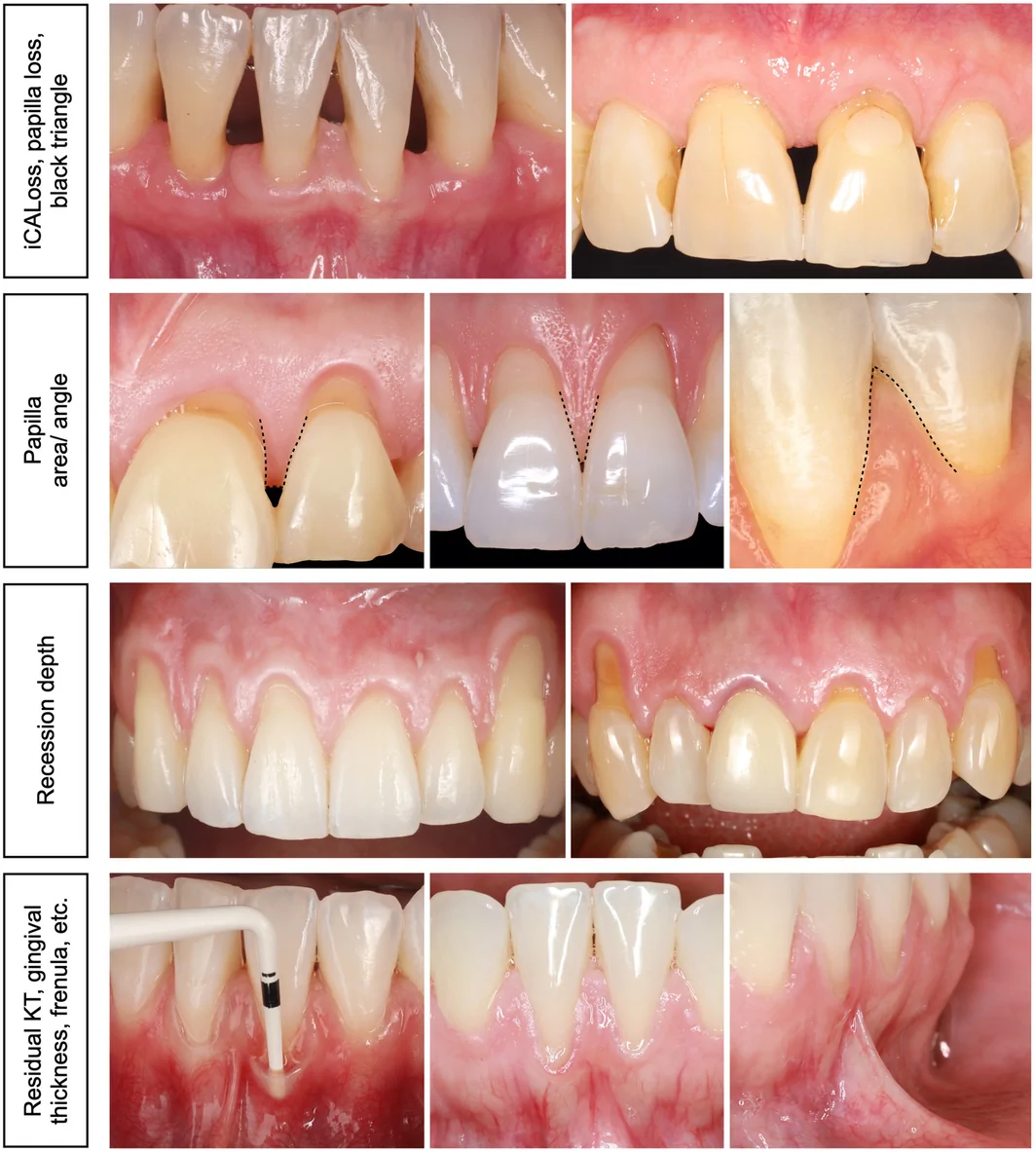
Key parameters affecting the complexity of root coverage procedures, the choice of the surgical technique, and the likelihood of obtaining complete root coverage (iCALoss, Interproximal clinical attachment level loss; KT, Keratinized tissue width).

CAF and TUN nonetheless differ in their surgical access and soft tissue handling. CAF, due to its open‐field design, offers superior visibility and maneuverability, which facilitates flap release, graft positioning, and coronal advancement [16, 26]. In contrast, tunnel approaches are valued for their minimal invasiveness and the preservation of the integrity of the papillae (particularly at deficient papillae and those with attachment loss), which helps maintain a superior vascular supply and minimizes the risk of postoperative scarring and flap sloughing [16, 27, 28, 29].

Each technique, however, presents certain technical challenges. CAF may be limited in sites where narrow or shallow papillae risk compromising vascularity, and early papillary sloughing or visible scarring may occur in some cases [16, 20, 28, 29, 30, 31]. TUN, although biologically advantageous due to the preservation of papillary integrity, provides more restricted access and can make flap release and graft stabilization more demanding.

It is thus reasonable to assume that certain clinical scenarios may benefit from a “combined” approach, whereby the elevation and access provided by CAF may be advantageous in some areas, while a TUN approach may be more appropriate in others. This rationale underpins the development of the concept of the Tunneled Coronally Advanced Flap (TCAF), a combined approach designed to leverage the strengths of these methods, based on site‐specific anatomical considerations [27, 29, 32, 33, 34, 35]. The TCAF approach advocates for a flexible, anatomically driven flap design. It involves a selective, papilla‐specific decision on whether to incise or preserve the papillae, and may incorporate one, two, or no vertical incisions, depending on the clinical indication. In essence, TCAF involves some areas that are opened and performed as a CAF, and other areas that are tunneled, either as a conventional TUN and/or as a modified tunneled approach (e.g., VISTA, IF‐VISTA, partial‐full‐thickness TUN, etc.) [36, 37, 38]. Whenever feasible, the number of vertical incisions should be minimized in the esthetic zone; however, the inclusion of one vertical incision during TCAF can aid in the tunnelization of flap segments, graft insertion, and flap release [27, 29, 32, 33]. The clinical factors guiding decisions on which papilla(e) to incise versus tunnel, and the corresponding TCAF design—whether an envelope flap or a design with one, two, or no vertical incisions—are discussed in detail in the following section.

Finally, it is important to emphasize that TCAF is conceptualized as a flexible technique rather than a rigid, predetermined protocol. Its purpose is to enhance presurgical critical thinking and encourage creative, anatomically based decision‐making. By tailoring flap design to local anatomical features, TCAF aims to optimize esthetic and clinical outcomes in challenging cases [27, 29, 32, 33].

## 3 Operational Definition and Components of the TCAF Framework

The TCAF is operationally defined as a hybrid root coverage flap design in which the decision to incise (open) or to tunnel (preserve intact while lifting it from the underlying bone) is made independently for each individual papilla included within the flap, based on its specific anatomical characteristics.

Three essential components must coexist for a flap to qualify as a TCAF. First, a papilla‐specific decision is made for every papilla within the flap, rather than applying a single, uniform approach to the entire surgical field. Second, the resulting flap must contain, simultaneously and within the same continuous surgical site, at least one open (CAF‐like) papilla segment and at least one tunneled (intact but lifted and separated from the underlying bone) papilla segment. It should be noted that connecting two flap areas apically to a papilla that has not been lifted from the underlying bone does not qualify as TCAF: every papilla within the flap must be either actively opened (incised and elevated) or actively tunneled (intact but lifted and separated from the bone), with no papilla left untouched in between. Third, the flap is coronally advanced, with the de‐epithelialized anatomical papilla(e) serving as the suturing bed at the opened sites.

Within this definition, several aspects of the technique can be modulated according to the clinical scenario. The number of vertical releasing incisions can be zero, one, or two, depending on the need for additional flap mobility, access, and rotation, and is generally minimized in the esthetic zone. The tunneled component may be performed as a conventional sulcular tunnel or, when indicated, may incorporate modified tunnel approaches, such as VISTA, IF‐VISTA, or partial–full‐thickness tunnel techniques [36, 37, 38, 39, 40, 41]. The thickness of flap elevation can also be tailored to the site, ranging from a fully split‐thickness preparation to a full‐thickness or a split‐full‐split design, depending on the soft tissue phenotype and whether the treated site involves teeth or implants.

It is important to clearly delineate the boundaries that separate TCAF from the two preexisting techniques from which it draws its principles. The conventional CAF, by definition, requires the incision, de‐epithelialization, and elevation of all the papillae adjacent to the recession defects included in the flap; no papilla is preserved as a closed tunnel. In particular, in the envelope CAF described by Zucchelli & De Sanctis [42], the apical portion of the flap is released through a partial‐thickness dissection extending beyond the distal ends of the horizontal incision [42]; in the envelope CAF for anterior teeth (“frontal approach”) [43], all papillae adjacent to the recession defects are incised and elevated, with only the inter‐incisive papilla left intact and the blade passed apically beneath its base to release the muscular fibers and frenular insertions [43]. In both variants, however, no papilla is tunneled, that is, lifted and separated from the underlying bone—the dissection occurs apical to the papilla base rather than beneath the papilla itself. TCAF is therefore conceptually and technically distinct, as it is defined by the co‐existence, within the same flap, of at least one incised/opened papilla and at least one papilla actively tunneled and lifted from the bone, with a papilla‐specific decision guided by site‐specific anatomical parameters. Conversely, a pure tunnel technique—whether conventional sulcular TUN, VISTA, IF‐VISTA, or any other modified tunnel variant [36, 37, 38, 39, 40, 41]—is defined by the integrity of every papilla within the surgical field, with no papilla being incised or opened. TCAF cannot be reduced to either of these two approaches: it is defined precisely by the simultaneous co‐existence, within the same continuous flap, of at least one opened (CAF‐like) papilla and at least one tunneled (closed) papilla. A flap in which all papillae are opened is, by definition, a CAF and not a TCAF; conversely, a flap in which all papillae are preserved intact is a tunnel technique and not a TCAF. This dual nature is what distinguishes TCAF as a distinct surgical entity rather than a simple modification of either parent technique, and what justifies its operational definition as a separate flap design within the broader spectrum of root coverage procedures.

## 4 Clinical Factors Affecting Flap Design

Numerous studies have identified critical factors influencing the success of root coverage procedures. In the context of CAF, key technical and anatomical parameters have been highlighted, including flap preparation, such as a “split‐full‐split” design versus a uniform split thickness elevation [44, 45], as well as flap mobility, stabilization, and suturing methods, all of which significantly impact the clinical outcomes [45, 46]. Anatomical variables such as interproximal bone and attachment levels [47, 48], papillae dimensions [8, 49, 50], tooth malposition [8], root prominence [49], gingival thickness [51], residual KT width apical and lateral to the recession defect [6], presence of non‐carious cervical lesions [49], defect size [52, 53], and tooth position within the arch [54, 55] have also been shown to influence the likelihood of achieving complete root coverage. Notably, several studies have identified a strong correlation between papillae height and mean root coverage (mRC) [49, 50, 53, 56], while papilla width has similarly been associated with greater root coverage following CAF [56]. According to Zucchelli et al., papillae dimensions are key prognostic indicators, influencing flap stability, vascular supply, and the overall outcomes of the surgical procedure [8]. Similarly, several factors have also been described relative to the outcomes of TUN techniques [57, 58, 59, 60, 61]. In particular, the severity of the gingival recession has been found to negatively impact both mRC and the likelihood of achieving CRC [59, 60, 62, 63].

Taken together, these findings suggest that CAF is most predictably executed in the presence of papillae with adequate width and height, whereas its effectiveness may be limited when treating sites with shallow or narrow papillae [8]. Conversely, TUN appears to offer advantages in overcoming interproximal attachment loss and deficient papilla morphology, expanding its utility in more papilla‐complex clinical scenarios [14, 31, 61, 62, 64, 65, 66].

In the authors' opinion, the primary anatomical considerations for the selection of the surgical approach and flap design should include:

- The papilla fill—which includes the extent of interproximal CAL loss and the presence or lack of papilla loss
- The papilla angle—which best reflects the dimensions of the papilla, encompassing both the width and the height of the interproximal soft tissue, and
- The depth of the recession defect—which conveys its severity.

Secondary factors, such as anatomical region, tooth malposition, presence of frenula, apical and lateral KT, and vestibular depth, may also influence the choice of technique and should be carefully assessed during presurgical planning (Figure 1) [6, 8].

### 4.1 The Papilla Fill

When interproximal clinical attachment level (CAL) loss, and/or papilla loss are present, particularly in sites with narrow papillae, preserving the integrity of the papillae is generally preferred. In these scenarios, the interproximal bone levels are typically apically positioned relative to their ideal location, which results in a compromised vascular supply to the papillae, and a more fragile papilla [8, 27, 44]. Elevating a flap by incising these papillae may further reduce perfusion, jeopardizing the vascular support needed for optimal healing of the anatomical papilla and adjacent interproximal tissues. This compromised vascularity increases the risk of flap sloughing during the early postoperative period. Sloughing of the flap at the interproximal regions may not only lead to fibrotic scar formation, but can also destabilize the flap at the midfacial areas, thus reducing the likelihood of achieving complete root coverage. Furthermore, vertical attachment loss and papillary deficiency represent inherent limitations for CAF, which relies on de‐epithelialization of the papillae and suturing of the flap at the papillary tip [8, 27, 44]. Conversely, tunneling these deficient papillae, whether with tunnel techniques or in a TCAF approach, offers several advantages. It facilitates flap mobilization and coronal advancement, while also allowing the insertion of a graft beneath the tunneled interproximal soft tissue to augment the papillae when needed. To ensure tension‐free coronal advancement at the level of the papillae, particularly when papillary enhancement is an additional goal, it is advisable to extend the tunneling approach to the palatal or lingual aspect as well [67, 68, 69, 70].

Based on the interproximal CAL loss and papilla fill, we suggest classifying papillae as flat, medium, or full (Figure 2). A papilla can be defined as “full” when the soft tissue completely fills the interproximal space, with no visible black triangle. In contrast, a papilla which is “flat” has its tip located at the same level as, or apical to, the cemento‐enamel junction (CEJ) of the adjacent teeth. Intermediate scenarios between “full” and “flat” are classified as “medium,” referring to papillae whose tip is located coronal to the CEJ of the adjacent teeth but that do not completely fill the interproximal space, resulting in a visible black triangle (Figure 2). It should be noted that papilla fill is assessed independently for each individual papilla and therefore cannot be replaced by the Cairo recession type classification [48], which is tooth‐based and reflects the comparative interproximal and mid‐buccal clinical attachment levels of the affected tooth rather than the morphology of each papilla.

**FIGURE 2:**
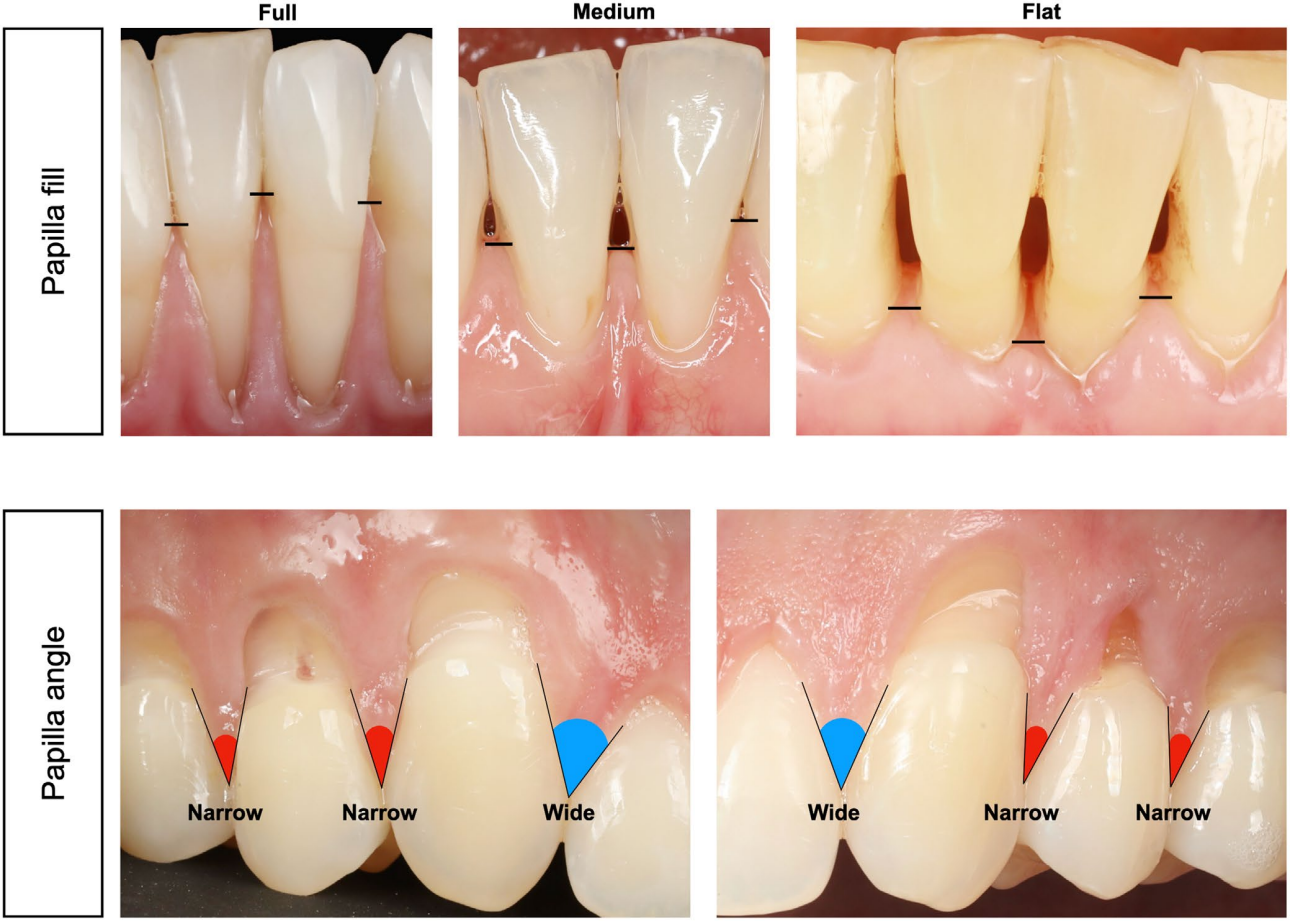
Clinical examples of papilla fill and papilla angle assessment. Based on papilla fill, a papilla is categorized into full, medium, or flat, while based on papilla angle, a papilla is defined as narrow or wide.

### 4.2 The Papilla Angle

Papillae with a narrow base, limited height, or both, are generally suboptimal in providing adequate vascular support to the flap, especially when incised, as these are likely to undergo significant “shrinkage” and be compromised further during the healing [8]. To characterize papillary morphology, the concept of papilla angle—which reflects both papillary height and width—can be utilized. Our group has recently observed that the papilla angle was significantly associated with tissue perfusion, such that an increasing papilla angle was significantly associated with higher tissue perfusion outcomes during the early healing (2–4 weeks) [Unpublished data]. In addition, flap sloughing at the 2‐week visit was also found to be associated with both papilla angle and presurgical tissue perfusion of the papilla [Unpublished data].

Papillae exhibiting narrow angles are generally considered less favorable for the CAF approach. In such cases, tunnelization of the papillae is preferred, as it can be less invasive to the anatomical structure and vascular integrity of the tissue. Proper tunnelization of narrow‐angled papillae can substantially reduce the risk of interproximal flap sloughing when compared to conventional incision and elevation techniques used in CAF. This aspect is particularly crucial not only with the use of autogenous connective tissue graft (CTG), but also when biomaterials and soft tissue substitutes are utilized. Based on our preliminary study and assessment, we have found that papillae can generally be classified based on a 30 degree angle threshold above which typically resembles adequate papillae and below which resembles narrower and weaker papillae (Figure 2).

### 4.3 The Depth of the Recession Defect

Regardless of the surgical approach or material(s) utilized, the literature consistently shows an association between gingival recession severity and likelihood of achieving complete root coverage (Figure 2) [44, 52]. Indeed, proper graft stabilization becomes even more critical in deep recessions, where the pronounced buccal bone dehiscence can impair graft nutrition and blood clot stability. In such cases, partial or, if needed, a full flap opening is generally preferred to ensure adequate access to the root surface and to facilitate proper graft stabilization, which can be achieved using the de‐epithelialized papilla(e), the periosteum, or the surrounding soft tissues. When managing multiple adjacent gingival recessions with varying degrees of severity, the TCAF approach typically involves incising one or both papillae adjacent to sites with more advanced recession defects. In such cases, one or two vertical incisions (at the distal ends of the flap) may also be incorporated to improve access, flap mobility, and rotation. Meanwhile, sites presenting with shallower recessions are often managed through a tunneled approach.

The design of papillary incisions at teeth with deeper and wider defects can vary depending on the overall flap configuration. If TCAF is performed as an envelope flap, an oblique incision may be used; alternatively, if a vertical incision is made near the papilla, a horizontal incision may be preferred. In either case, the orientation of the incision(s) should be thoughtfully planned in alignment with the intended direction of flap advancement and stabilization to support optimal adaptation and tension‐free coronal positioning.

### 4.4 Standardized Assessment of the Decision Tree Parameters

To support reproducibility in both clinical application and future research, a standardized assessment protocol is proposed for each of the three parameters that drive the decision tree

- Papilla fill is classified visually as full, medium, or flat, based on the position of the papilla tip relative to the apical end of the contact point. A papilla that completely fills the interproximal space, with no visible black triangle, is defined as full. A papilla whose coronal tip is located at the level of the CEJ of the adjacent teeth is defined as flat. Intermediate situations, in which the papilla tip lies coronal to the CEJ but does not reach the apical end of the contact point—resulting in a visible black triangle—are defined as medium (Figure 2).
- Papilla angle is measured on standardized clinical photographs taken perpendicular to the long axis of the papilla to be analyzed; the angle is defined as the one whose vertex is located at the tip of the papilla and whose two sides run along the mesial and distal lateral borders of the papilla up to the level of the CEJ of the two adjacent teeth and is quantified using digital angular measurement software (Figure 2). A papilla is classified as narrow when the angle is ≤ 30° and as wide when the angle is > 30°.
- Recession depth is measured in millimeters with a UNC‐15 periodontal probe from the CEJ to the midfacial gingival margin of the affected tooth, as extensively described in the literature [71, 72, 73, 74].

These standardized assessments should be performed during the presurgical evaluation and are intended to ensure consistent application of the decision tree across operators and centers, while acknowledging that the specific thresholds remain hypothesis‐generating and require prospective validation.

## 5 Papilla‐Based Decision Tree for Selecting an Open or Tunneling Approach for Each Papilla According to Site‐Specific Characteristics

Based on the anatomical factors and clinical characteristics of gingival recessions discussed previously, a novel papilla‐based decision tree for root coverage procedures is presented in Figure 3. This framework is intended to guide clinicians in tailoring interventions to individual patient needs and site‐specific anatomical considerations by providing recommendations on opening/incising or tunneling for each papilla.

**FIGURE 3:**
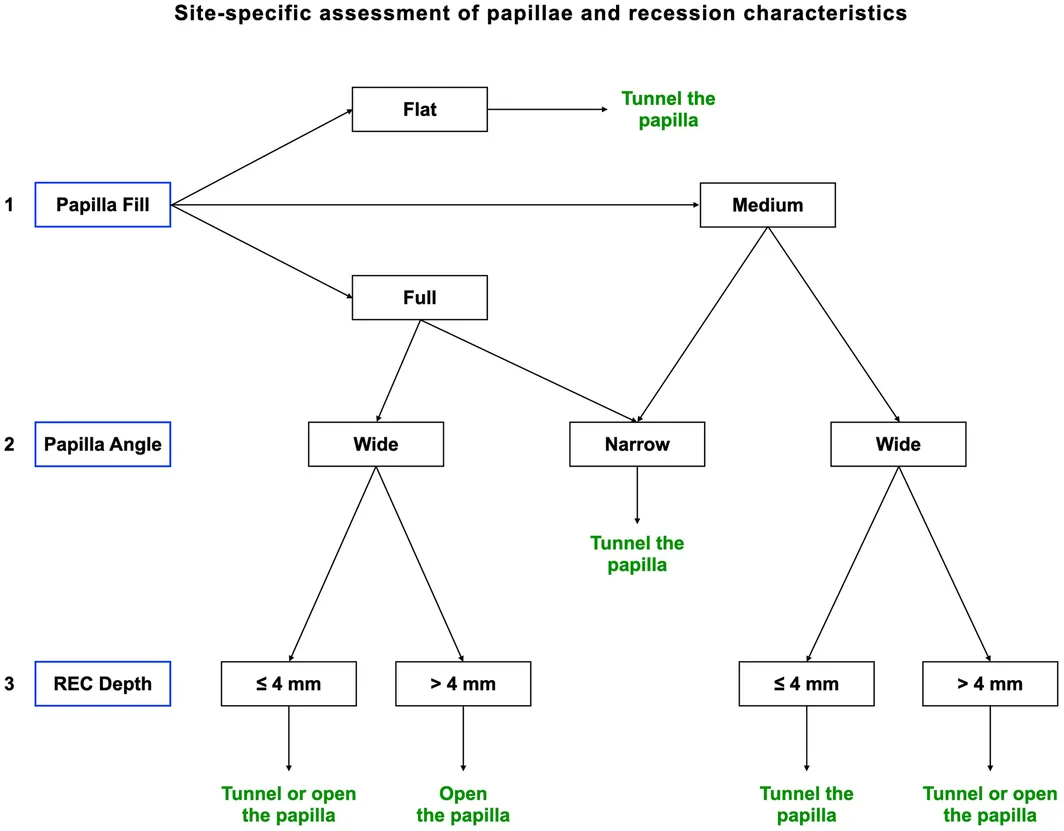
Papilla‐based decision tree guiding the choice between an open or tunneling approach for each individual papilla according to site‐specific papilla and recession characteristics. The decision tree is proposed as a hypothesis‐generating, experience‐based clinical aid: prospective validation is required before its nodes and thresholds can be considered established clinical criteria. The recommendations are therefore intended as clinical guidance rather than absolute rules, and exceptions may be applied based on operator judgment and the specific clinical scenario.

Key decision‐making parameters include: (1) Papilla fill, (2) Papilla angle, and (3) Recession depth. The first decision node is represented by Papilla fill, which is also correlated to the interproximal CAL. It is recommended not to open a flat papilla, as it is associated with substantial CAL loss and may benefit from a tunnellike approach that can also allow for the reconstruction of the interproximal soft tissue. On the other hand, to determine whether sites characterized by full or medium papilla fill should be opened or tunneled, we have to go to the decision node #2 of the decision tree, involving the assessment of the Papilla angle. Papillae with narrow angle, regardless of the papilla fill, should also not be opened, while for determining the decision making for wide papilla (either with a full or a medium papilla fill), we should move to the third and last decision node, which is based on recession depth.

The assessment of the severity/depth of the recession (node #3) is related to the clinical decision making on whether one or more access through papilla/ae opening could be favorable for stabilizing the graft around a challenging recession defect. When the papilla fill is either full or medium, the angle is wide and the recession depth is > 4 mm, it is overall recommended to open the papilla, as its elevation and management, as described for CAF. This selective papilla opening can facilitate the surgical procedure, with the surgical papilla that has relatively high chances of healing by primary intention without sloughing when sutured over the wide de‐epithelialized anatomical papilla. Nevertheless, readers should bear in mind that these recommendations do not imply that TUN procedures are contraindicated at sites with recession depth > 4 mm, rather that opening one more papilla of adequate dimensions in these scenarios may be beneficial.

In presence of recession depth ≤ 4 mm, papillae characterized by medium papilla fill and wide angle should not be opened, while papillae characterized by full papilla fill and wide angle may be opened or may be tunneled, depending on the overall needs and strategies. For example, opening a full and wide papilla in presence of a recession depth ≤ 4 mm may be beneficial if the goal is not only to cover the tooth but also to augment the adjacent papilla.

When reading this papilla‐based decision tree, clinicians should keep in mind that these are clinical suggestions based on anatomical characteristics and theoretical likelihood of obtaining healing by primary intention, but ultimately, the choice of flap design, and whether to open or not each papilla, should also take into account anatomical region, tooth malposition, presence of frenula, residual KT, vestibular depth, and surgeon's experience. In addition, the specific numerical thresholds proposed in the decision tree (a papilla angle of 30° and a recession depth of 4 mm) are intended as practical guideposts derived from the authors' clinical experience and require prospective validation. Clinicians are therefore encouraged to focus on the underlying anatomical concepts—namely, the recognition of a narrow versus wide papilla and of a shallow versus deep recession—rather than on the rigid application of the exact numerical cut‐offs.

## 6 Surgical Technique and Execution of the TCAF

The following section outlines the general surgical steps and principles for executing the TCAF. While specific details may be adapted according to the clinical scenario, the general framework described below reflects the authors' approach and is intended to improve reproducibility and educational value. The technique has been described in detail and illustrated in previously published case reports and case series by the authors [27, 29, 32, 33, 34, 35], to which the reader is referred for step‐by‐step photographic documentation.

### 6.1 Presurgical Planning and Flap Design

Before surgery, the clinician should apply the papilla‐based decision tree (Figure 3) to each papilla within the planned flap, classifying it according to papilla fill, papilla angle, and recession depth, and establishing for each papilla whether it will be opened (CAF component) or tunneled (TUN component). The planned flap design—envelope, single vertical incision, or two vertical incisions—and the position of any releasing incisions should also be determined at this stage. Standardized preoperative photographs should be obtained perpendicular to the arch to allow quantification of papilla angles using digital software, as described in Section 4.4.

### 6.2 Sequence of Flap Preparation

The authors recommend initiating the surgical procedure with the TUN component, followed by the CAF component, and placing any vertical releasing incisions last. This sequence is preferred for several reasons. Beginning with the tunnel preserves tissue integrity while the field is undisturbed and the blood supply is uncompromised, facilitating atraumatic instrumentation beneath the papillae designated for tunneling. Completing the CAF component thereafter allows the clinician to connect the tunneled and open segments of the flap and to verify adequate coronal mobility across the entire flap. Vertical incisions, when included, are made last to further mobilize the flap and improve rotation, once the extent of additional release required has been assessed. Nevertheless, this sequence should be considered a practical recommendation rather than a strict requirement. Currently, there is no evidence indicating that initiating the CAF component before the TUN component results in inferior clinical, esthetic, or patient‐reported outcomes. Consequently, the order of flap preparation may be modified according to the clinician's preference and the specific demands of the case.

### 6.3 Tunnel (TUN) Component

The tunnel component begins with intrasulcular incisions made using a microsurgical tunnel blade along the buccal aspect of each tooth to be tunneled. The tunneling flap preparation is then initiated by inserting either a microsurgical tunnel blade or a straight tunneling knife through the sulcus, aiming to create a full thickness tunnel. When microsurgical tunnel blades are used, the dissection is often performed in a deep split thickness plane, leaving most of the tissue thickness within the flap and thereby closely resembling a full thickness tunneling approach. This allows elevation of the keratinized tissue from the underlying structures. After passing the mucogingival junction, the tunnel preparation transitions to a split thickness dissection to ensure sufficient flap mobility for tension‐free coronal advancement.

Once tunneling has been performed at the midfacial aspect of all intended sites, the interproximal regions are connected through a full thickness tunnel, or through a deep split thickness tunnel closely resembling a full thickness preparation, using an angulated tunneling instrument or an angulated microsurgical tunnel blade. At this stage, the papillae designated for the CAF component remain untouched and have not yet been tunneled. Following further release of the apical portion of the flap, performed either with tunneling blades or with a 15C blade inserted through the CAF opened regions, attention can be directed toward the papillae to be released.

The complete detachment of the papilla from the underlying bone, which conceptually and technically distinguishes tunneling of a papilla from simply connecting two flap areas apical to an intact papillary base, is achieved as follows. First, an intrasulcular dissection is extended into the proximal aspect of the tooth using a papilla elevator, thereby separating the papillary borders from the adjacent teeth. The same instrument is then positioned at the base of the papilla and gently advanced from the base toward the tip, separating the entire papillary soft tissue complex from the underlying interproximal bone. This maneuver is performed with the instrument oriented parallel to the interproximal bone surface, using careful sweeping movements to avoid perforation of the papillary tip.

When papilla augmentation is an additional objective, the dissection is extended to the palatal or lingual aspect, ensuring complete 360‐degree mobilization of the papilla. The papilla is thus elevated and separated from the underlying bone in its entirety, remaining as an intact sleeve of soft tissue while being fully mobile and detached from its osseous bed. In contrast, simply connecting flap segments apical to the papillary base without performing this complete subpapillary detachment does not constitute papilla tunneling and does not provide the same degree of flap mobility, vascular redistribution, or capacity for graft insertion beneath the papilla. Therefore, such an approach should not be considered a TCAF procedure.

### 6.4 CAF Component

Papillae designated as open (CAF component) are managed following the principles of the coronally advanced flap, as described by Zucchelli and De Sanctis [26, 42, 43]. In the TCAF, at least one papilla is elevated either through an oblique incision, made through the surgical papilla at an angle aligned with the planned direction of coronal advancement, or through a horizontal incision, which is usually followed by a slightly divergent vertical releasing incision. The papilla is then elevated in a split‐thickness manner, while the remaining interproximal coronal soft tissue (“anatomical papilla”) is de‐epithelialized using a microsurgical blade and/or microsurgical scissors to create a vascular connective tissue bed for flap stabilization.

The flap is elevated following a split‐full‐split design, with split‐thickness dissection at the papillary and marginal tissue level, transitioning to full‐thickness periosteal elevation in the middle portion of the tooth apical to the gingival recession, and returning to a split‐thickness plane apical to the mucogingival junction to allow tension‐free coronal advancement.

The open and tunneled segments of the flap are joined by extending the full thickness dissection or tunnel preparation from the CAF opening toward the tunneled portions of the flap, or vice versa, thereby creating a continuous single piece flap across the entire surgical field. Particular attention should be paid to ensuring that all tunneled areas are uniformly connected and free of residual fibrous attachments that could interfere with graft passage, positioning, and adaptation beneath the flap.

### 6.5 Graft Insertion and Stabilization

When a CTG or a soft tissue substitute is used, graft insertion is facilitated by the openings created at the incised papillae and, when present, by the vertical releasing incision(s). Unlike conventional tunnel techniques, where the graft is typically advanced into position using traction sutures, the open segments of the TCAF provide direct visualization and access for graft placement, allowing more controlled and precise positioning. The graft(s) should extend beneath both the open and tunneled segments of the flap, including beneath the tunneled papillae when papilla augmentation is intended. Graft stabilization is achieved by suturing the graft to the flap within the tunneled regions and to the de epithelialized anatomical papilla(e) and/or the periosteum within the CAF prepared regions, depending on the graft type and the specific clinical situation.

### 6.6 Flap Advancement and Suturing

Coronal advancement of the flap should be verified before suturing to confirm tension free positioning coronal to the CEJ across the entire surgical field, including the tunneled interproximal areas. The suturing strategy in TCAF reflects its hybrid nature and differs between the open and tunneled components. At the open (CAF) papillae, the surgical papilla is advanced coronally and sutured over the de‐epithelialized anatomical papilla using sling sutures, aiming for passive, tension free adaptation [26]. The tunneled papillae can be coronally advanced and stabilized either with sling sutures [27, 75] or with suspensory sutures anchored to flowable composite placed to close the contact points [36, 39, 76, 77]. When present, simple interrupted sutures at the vertical releasing incisions provide additional stabilization of the mobilized flap segments. A Cornick suture may also be placed apical to the tunneled portion of the TCAF flap to increase flap verticality and vestibular depth, while promoting closer adaptation of the flap to the underlying periosteum [36].

## 7 Clinical Applications of the TCAF

The most common indications and clinical applications of the TCAF approach are summarized in the Table 1, and illustrated in Figures 4, 5, 6.

**TABLE 1:** Common case scenarios where the TCAF can be applied and the corresponding rationale.

Case scenario	Rationale for the TCAF
Multiple GRs with interproximal CAL loss or not ideal papillae angle	Tunnelization of the papillae associated with CAL loss or not ideal papilla angle, with one or more “openings” that are performed at the papillae with adequate height and angle, to facilitate the tunnelization, the release of the flap, and the insertion of the graft in the desired position
Isolated GRs with one deficient papilla/one papilla with CAL loss	The papilla with adequate height and angle is elevated to have access, allowing for an easier flap release and graft stabilization
Root coverage at rotated and/or malpositioned teeth	Rotated and/or malpositioned teeth have often one or both deficient papillae. Incising and opening the deficient papilla(ae) have a high risk of flap sloughing. The TCAF can reduce this risk by maintaining the integrity of the deficient papilla (ae), while providing access for the management of the flap and the stabilization of the graft
Treatment of multiple and “uneven” GRs in the esthetic zone	Performing a conventional CAF would require the creation of “false” recessions, with scalloping incisions, that may result in keratinized tissue loss. The TCAF allows to open the flap only at the most severe sites, while tunneling the teeth with shallow recessions, or without recessions. Most of the region of the flap are tunneled, with one or more papillae that are incised and one or more vertical incisions that are commonly performed distal to the canine(s) or premolar(s), when possible, to increase access, visibility, and ease in flap release and graft stabilization, without the risk of impairing the esthetic outcomes due to postoperative scar formation
Minimally invasive envelope approach	When performing root coverage at multiple teeth, a minimally invasive envelope flap (e.g., opening two papillae in the middle of the flap only) can be performed to facilitate graft insertion, while reducing flap opening and thus risk of papilla sloughing, scars, and, possibly, postoperative morbidity
Root coverage in the lower anterior region maintaining/increasing the vestibular depth	Root coverage procedures in the lower anterior region have often the risk of reducing the vestibular depth. Adding one or two verticals in the flap, while tunneling deficient and not ideal papillae, allows to combine the benefits of tunnel technique, while maintain the verticality of the flap and reducing the chances of losing vestibular depth. The vertical incisions also facilitate the removal of the labial submucosal tissue and an adequate flap release
Root coverage in the mandibular premolar region reducing the risk injury to the mental nerve	Performing one or more opening(s) allows to release the flap at its most coronal and superficial regions compared to TUN, reducing the risk of provoking injuries to the mental nerve
Treatment of lingual gingival recessions	Lingual recessions can be treated with an envelope TCAF with incisions and opening of the anterior papillae, with tunnelization of the posterior lingual region, to avoid vertical incisions and injuries to the lingual anatomical structures
Simultaneous root coverage and papilla augmentation	The TCAF can provide access to deficient papillae by opening one or more adjacent papillae with adequate height and width. The papillae to be augmented are then tunneled, allowing placement of one graft beneath the papillae over the bone crest, and a second graft on the buccal aspect for root coverage
Treatment of combined defects at teeth and implant sites and papilla augmentation	The TCAF allows to select a site‐specific papilla tunnelization or opening, together with a site‐specific flap elevation (split‐thickness vs. full‐thickness) that is particularly crucial when treating healthy implants in the anterior region exhibiting esthetic complications and adjacent teeth with gingival recessions. This approach also allows for simultaneous soft tissue grafting underneath the papillae for augmenting the interproximal soft tissue

Abbreviations: CAL, clinical attachment level; GRs, gingival recessions; TCAF, Tunneled Coronally Advanced Flap.

**FIGURE 4:**
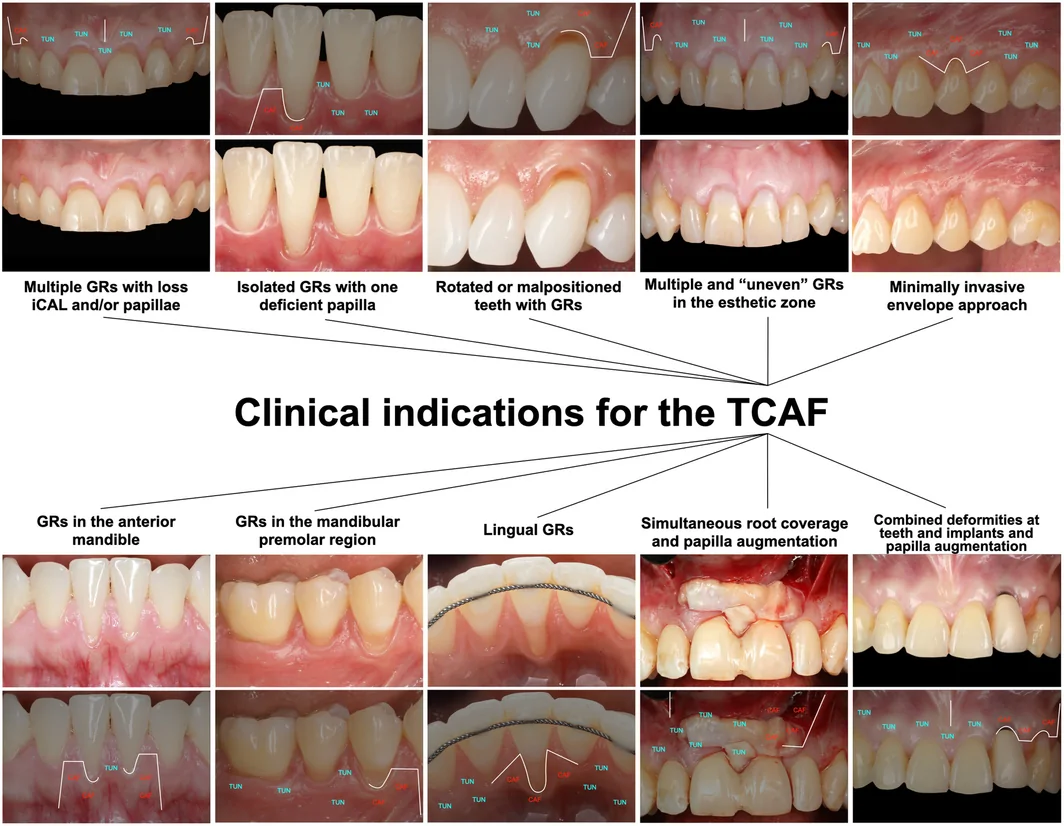
Clinical indications for the Tunnel Coronally Advanced Flap (TCAF) (CAF, coronally advanced flap; GRs, gingival recessions; iCALoss, interproximal clinical attachment level loss; TUN, Tunnel technique). Note that the TCAF design may or may not include vertical incisions, and may or may not include areas tunneled through the VISTA [38] or modified VISTA approaches [36, 37].

**FIGURE 5:**
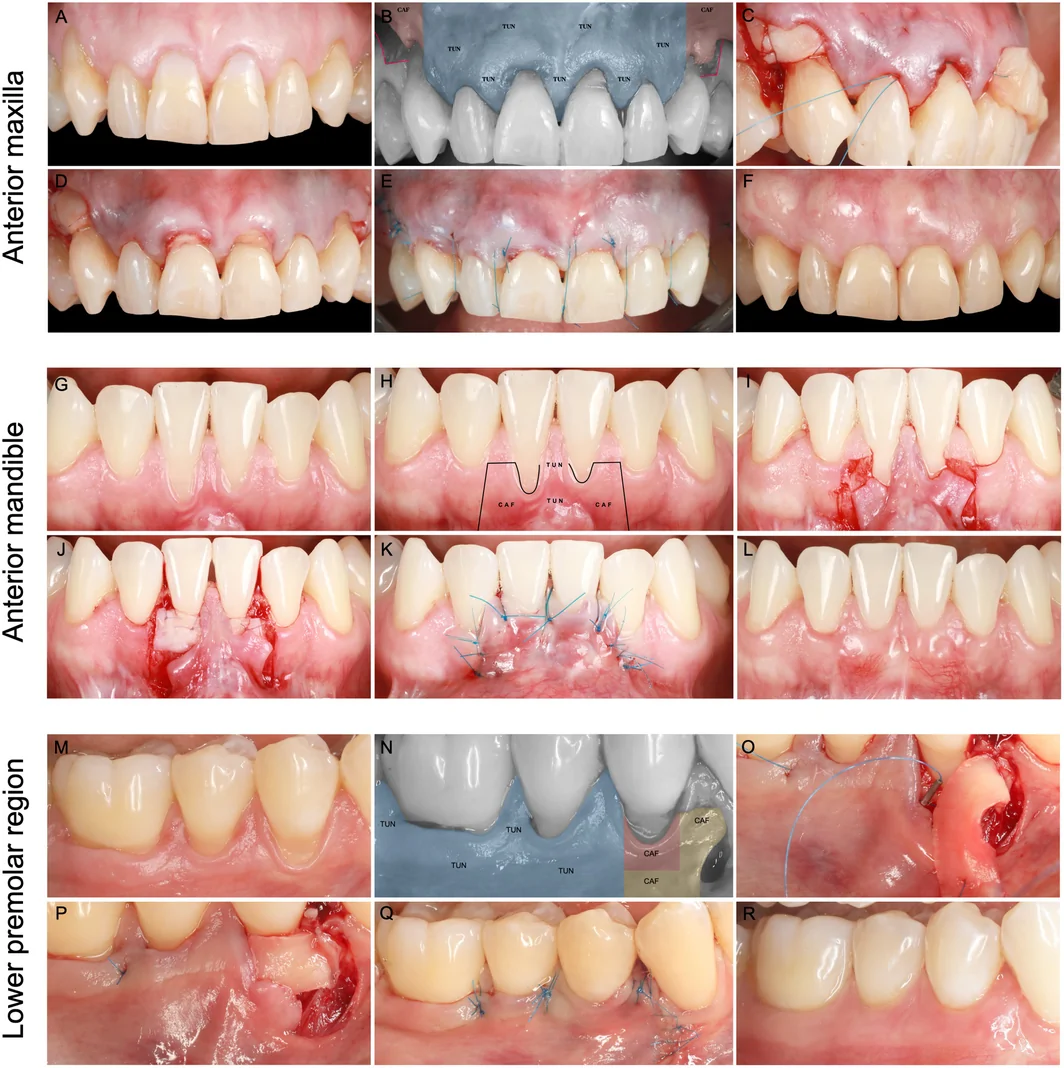
Clinical examples of root coverage procedures using the Tunnel Coronally Advanced Flap (TCAF) in combination with connective tissue graft for the treatment of multiple gingival recessions in the anterior maxilla (A–F), anterior mandible (G–L), and lower premolar region (M–R).

**FIGURE 6:**
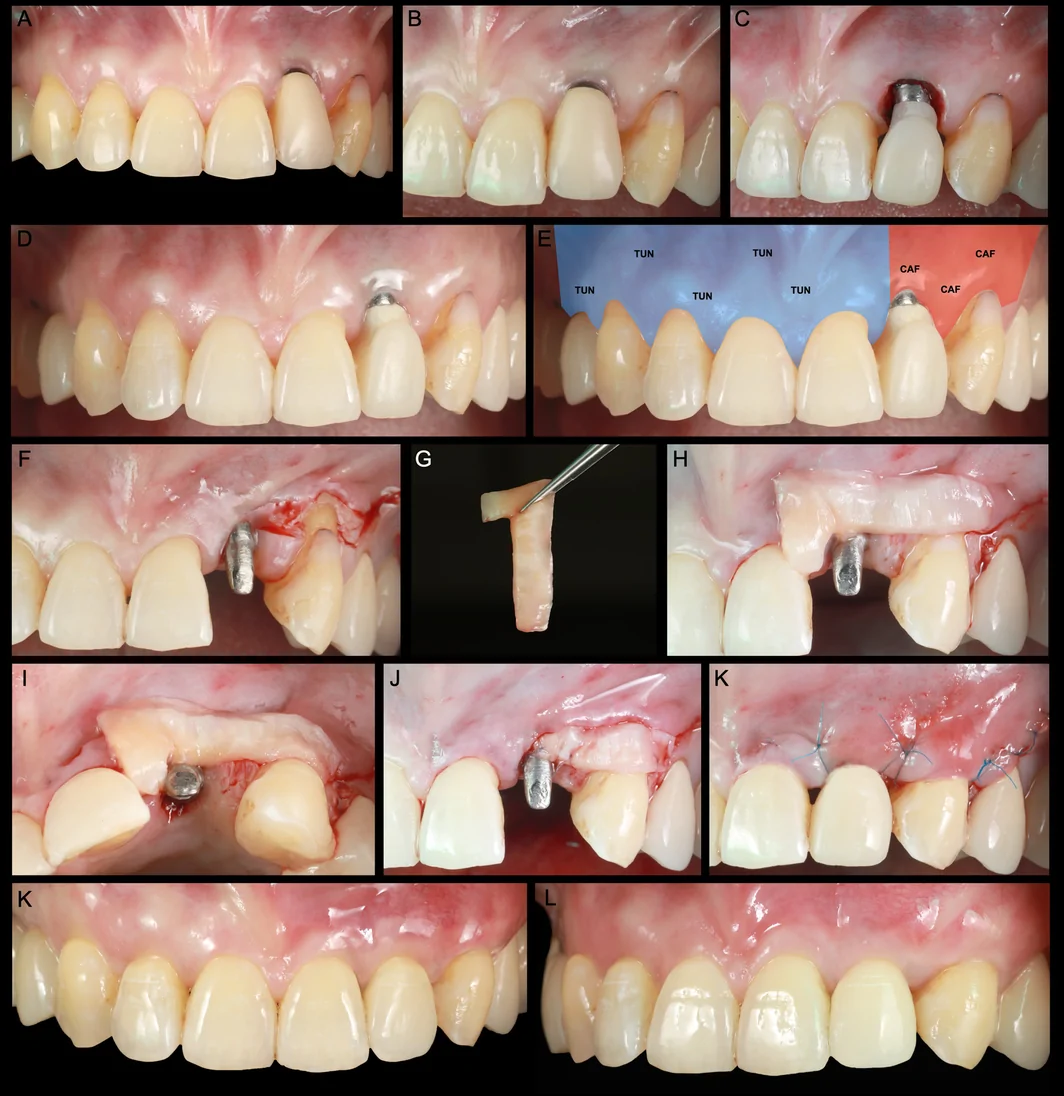
Treatment of combined soft tissue deformities involving multiple teeth and a single dental implant using the Tunnel Coronally Advanced Flap (TCAF) in a patient with esthetic concern. (A, B) Baseline presentation with the anterior teeth exhibiting gingival recessions, and the left lateral incisor presenting with a peri‐implant soft tissue dehiscence (PSTD). (C) A presurgical prosthetic treatment was performed, fabricating a new implant‐supported temporary crown. (D) After 1 month, the surgical intervention was performed. (E) TCAF design. (F) Split‐thickness CAF was executed on the midfacial aspect of the implant. (G–I) L‐shaped connective tissue graft. (J) One portion of the graft was positioned beneath the tunneled mesial papilla of the implant to enhance its height and overall volume, while the longer portion of the graft was sutured on the buccal aspect of the implant and the canine; the previous cervical restoration on the canine had been removed beforehand. (K) Flap suturing. (K, L) One‐year result, demonstrating complete resolution of the soft tissue deformities. The implant was restored with a new definitive implant‐supported crown, and the left canine was rehabilitated with a cervical resin restoration.

In brief, TCAF may be particularly advantageous in the treatment of multiple adjacent gingival recessions with some teeth showing interproximal CAL loss and flat and/or narrow papillae. In these cases, the ability to minimize invasiveness to the already compromised papillae via tunnelization, while selectively creating openings for flap release, addresses limitations associated with conventional sulcular TUN, especially in accessing the most apical regions, which can be technically demanding and time‐consuming.

These selective, site‐specific incisions, performed with or without vertical releasing incisions, not only improve flap mobility but also simplify graft insertion. Compared to conventional TUN, where the graft is often pulled into place using sutures, TCAF allows for a faster, more controlled, and more precise graft placement. In addition, the “openings” allow for lateral and apical tunneling, as opposed to sulcular TUN alone, which also reduces invasiveness to the already fragile and thin sulcular margins of the teeth to be treated. This rationale also applies to the management of isolated gingival recessions involving a single deficient papilla, such as those found at rotated or malpositioned teeth [8], and to minimally invasive, esthetically sensitive procedures in the anterior maxilla, where the TCAF maintains control over flap release and graft stabilization, while minimizing visible incisions.

In cases involving multiple or “uneven” gingival recessions, especially in the esthetic region, TCAF seems to be particularly well‐suited. The technique permits selective elevation of flap segments using CAF principles at sites with severe recession, while adjacent shallow defects, or areas with no recession, can be treated with a tunneled approach. This reduces the risk for scar formation and also avoids the need for submarginal incisions at unaffected sites, which may potentially reduce the width of keratinized tissue. TCAF, therefore, offers a balanced, anatomy‐driven approach, optimizing both function and esthetics.

In the anterior mandible, root coverage procedures are often complicated by anatomical challenges such as a thin soft tissue phenotype, frenal and muscle pull, shallow vestibule, and narrow papillae [54, 55, 61, 78]. Preserving the integrity of the narrow papillae through tunneling can be facilitated by creating horizontal and/or vertical incisions at distal papillae, when dimensions and angles are adequate. These incisions improve access and visibility, allowing for effective tunnelization of deficient/narrow papillae, flap release, and removal of restrictive labial submucosal tissue. This configuration supports precise graft insertion and stabilization to both the de‐epithelialized papillae and the periosteum. TCAF also enables coronal advancement of opened papillae and secure stabilization of the flap via periosteal sutures placed at vertical incisions.

Additionally, TCAF allows for the placement of CTG and/or biomaterials not only along the buccal surface but also beneath intact, tunneled papillae. This offers a unique opportunity for simultaneous root coverage and papilla augmentation (Figure 6).

## 8 Limitations

This manuscript presents a conceptual framework and clinical decision tree based on the authors' experience and preliminary unpublished data. Several limitations should be acknowledged. First, the papilla‐based decision tree, while grounded in anatomical principles and clinical observation, has not yet been prospectively validated in a controlled clinical trial. Readers should keep in mind that the specific thresholds proposed (angle of 30° discriminating narrow from wide papillae, and 4 mm of recession depth suggesting flap opening vs. tunnelization) are derived from preliminary data and clinical experience rather than large‐scale, multicenter studies and should therefore be interpreted with caution.

Second, the decision tree was deliberately built around three primary parameters (papilla fill, papilla angle, and recession depth) to remain applicable in daily practice, and does not explicitly incorporate every anatomical variable that may influence root coverage outcomes. Additional constraints, such as apical and lateral KT, recession width, non‐carious cervical lesions, gingival thickness, vestibular depth, and presence of frenula, should be carefully assessed during presurgical planning and may, in selected cases, modify the recommendations derived from the decision tree.

Third, the TCAF is operator‐dependent and technique‐sensitive. The selective tunnelization of narrow or compromised papillae, in particular, requires careful instrumentation and adequate flap mobilization to preserve papillary vascular supply, and the associated learning curve may be steeper than that of conventional CAF. Variability in outcomes across operators with different levels of experience is therefore expected, and data on clinician‐reported complexity and patient‐reported outcomes remain limited.

Finally, the statements presented in this manuscript are not all supported by the same level of evidence. Statements regarding the efficacy of CAF and tunnel techniques in their respective ideal indications and the prognostic role of papilla dimensions, interproximal clinical attachment level, recession depth, gingival thickness, and keratinized tissue width are evidence‐based, being supported by the cited randomized trials and systematic reviews. Statements regarding the biological advantage of preserving papillary integrity in deficient papillae and the rationale for combining open and tunneled segments within the same flap are biologically plausible but not yet confirmed by dedicated comparative trials of the TCAF. Statements regarding the specific thresholds, the recommended TCAF designs in the decision tree, and the clinical indications summarized in Table 1 are expert‐opinion‐based, reflecting the authors' clinical experience and the integration of available evidence into a coherent decision‐making framework.

Future randomized clinical trials should compare the TCAF, guided by the papilla‐based decision tree, with conventional CAF or tunnel techniques across different clinical scenarios, with particular attention to sites with deficient papillae, moderate to advanced interproximal attachment loss, or uneven recession depth among adjacent teeth. Multicenter studies evaluating the TCAF in diverse populations and anatomical regions are also needed to validate the proposed thresholds, characterize the learning curve, and capture patient‐reported outcomes. Studies exploring the application of the TCAF at adjacent teeth and implants, and for combined root coverage and papilla augmentation, are similarly encouraged.

## 9 Future Directions of the TCAF

TCAF may also be valuable in the treatment of multiple soft tissue deformities involving both natural teeth and dental implants with peri‐implant soft tissue dehiscences (PSTDs) and deficient papillae, or those with disease [79, 80, 81]. These cases often require highly individualized, site‐specific approaches involving variable combinations of papilla incision or tunnelization and tailored full‐thickness or split‐thickness flap designs. While a split‐full‐split CAF is typically performed for gingival recessions around teeth [26], a completely split‐thickness flap is preferred at healthy implant sites to preserve connective tissue fibers in cases of buccal bone dehiscence [82, 83]. In such complex cases, TCAF offers a flexible framework that accommodates these site‐specific anatomical and surgical requirements, supporting PSTD coverage and phenotype modification while also enabling simultaneous peri‐implant papillae augmentation (Figure 6) [79]. The use of TCAF for augmenting vertical soft tissue height and papillae has recently been reported by our group and termed vertical TCAF (verTCAF) [79].

Lastly, a TCAF approach may also be beneficial when performing periodontal regenerative therapy at sites with gingival recession defects, where the access is provided by opening one or more papillae adjacent to the one with the infrabony defect, which, conversely, is tunneled and often also augmented with a soft tissue graft [68, 84, 85]. For this application, the technique was first described and named Coronally Advanced Entire Papilla Preservation Flap by Rasperini and colleagues [68].

## 10 Concluding Remarks

This manuscript has outlined the clinical rationale for the Tunneled Coronally Advanced Flap (TCAF), a versatile surgical approach and treatment philosophy conceptualized in response to the specific anatomical indications of each individual case. Due to its flexibility and papilla‐specific opening or tunnelization, the TCAF is suitable for a wide range of clinical situations.

To support clinicians in surgical planning, a novel papilla‐based decision tree was introduced, presenting recommendations for papilla opening or tunnelization, based on three key parameters: papilla fill, papilla angle, and recession depth. Nevertheless, clinical studies are needed to validate these recommendations and establish the optimal indications for TCAF in various clinical scenarios.

## Author Contributions

L.T., and S.B. contributed to the initial idea, manuscript outline, manuscript writing and clinical cases. L.T. and S.B. were involved in the initial and final drafting of the work, and in the critical manuscript reviewal, and are accountable for all aspects of the work.

## Conflicts of Interest

The authors declare no conflicts of interest. The Tunneled Coronally Advanced Flap (TCAF) represents a surgical concept developed by the authors based on clinical experience and existing principles in periodontal plastic surgery.

## Data Availability

Data sharing not applicable to this article as no datasets were generated or analysed during the current study.
